# Understanding the role of damping and Dzyaloshinskii-Moriya interaction on dynamic domain wall behaviour in platinum-ferromagnet nanowires

**DOI:** 10.1038/s41598-017-04088-8

**Published:** 2017-07-04

**Authors:** J. Brandão, S. Azzawi, A. T. Hindmarch, D. Atkinson

**Affiliations:** Department of Physics, Durham University, Durham, DH1 3LE United Kingdom

## Abstract

Heavy metal layers, exemplified by Pt, are known to play a significant role in the magnetization behaviour of thin-film ferromagnets by three distinct mechanisms that can each contribute to the reversal process. These include modifying the local magnetization state via an interfacial Dzyaloshinskii-Moriya interaction (IDMI), enhancement of the damping, via *d-d* hybridisation and spin-pumping across the interface, and the mediation of the magnetization switching, with the flow of current through a system, via the spin-Hall effect. Here we show for a system with weak interfacial DMI (NiFe/Pt) that the measurement of magnetic field-driven magnetization reversal, mediated by domain wall (DW) motion, is dominated by the enhanced intrinsic damping contribution as a function of the Pt capping layer thickness. But, we also show micromagnetically that the IDMI and damping also combine to modify the domain wall velocity behaviour when the damping is larger. It is also noted that Walker breakdown occurs at lower fields and peak DW velocity decreases in the presence of IDMI. These results highlight the significance of the relative contributions of the damping and the IDMI from the heavy metal layer on the magnetization reversal and provide a route to controlling the DW behaviour in nanoscale device structures.

## Introduction

Heavy metal layers in contact with ferromagnetic materials have been shown to lead to several remarkable phenomena associated with large spin-orbit coupling that can modify the micromagnetic state of the magnetization, enhance the damping of magnetization precession and drive magnetization reversal via the propagation of pure spin-current generated by the spin-Hall effect in the heavy metal layer^1–6^. In a given system these phenomena can all occur and the route to improved functional performance for spintronics-based device applications requires an understanding of the effective contributions of each of these physical mechanisms.

The interfacial Dzyaloshinskii Moriya interaction emerges from anti-symmetric exchange that was predicted to have a notable effect at the NM/FM interface^7–11^. The strength of the IDMI depends upon the materials involved with a larger IDMI in Co/Pt bilayers compared to the NiFe/Pt system^12, 13^, although a range of values have been reported for both^12, 14–16^. Though smaller in magnitude than the direct ferromagnetic exchange interaction, IDMI can modify the local magnetic orientation and dynamic magnetization processes can thus be affected by this IDMI contribution.

The large spin-orbit interaction of the heavy metal layer affects the damping of precession in the ferromagnetic layer, leading to a significant increase in damping^3, 5, 6, 10^. The damping enhancement occurs via *d-d* hybridisation at the interface, which locally modifies the damping, and by a process of ‘spin-pumping’, where precessionally driven spin-waves propagate from the ferromagnet across the FM/NM interface and are dissipated in the heavy metal layer, giving rise to increased damping in the system^3, 5, 6, 17, 18^. The combination of the two effects is responsible for increasing the overall intrinsic damping. Moreover, extrinsic damping due to two-magnon scattering associated with inhomogeneities may also play a role depending upon the nonuniformity of the interfacial structure. Usually, the precession and damping depend on the thickness of the heavy metal layer. For instance, the damping of Co shows a rapid increase with initial coverage of Pt and then tends to a plateau for thicknesses beyond 1 nm^3, 6^.

The spin-Hall effect has also been widely explored in NM/FM systems^11, 19, 20^. In Co/Pt-based device structures the flow of charge current creates a spin-current in the heavy metal that can propagate across the interface into the ferromagnet to move magnetic domain walls in the FM layer^4, 11^. The interface has been shown to play a role in the efficient functionality of this process in relation to the magnitude of spin Hall angle (SHA)^11, 19, 20^.

However, although the mechanisms occurring at the NM/FM interface have been explored in magnetic wires with strong IDMI and perpendicular anisotropy, few studies have addressed the influence of the heavy metal layer on in-plane magnetization processes and in nanowires^21, 22^. Furthermore, whilst it is known from experimental and micromagnetic studies of in-plane magnetic nanowires that the field driven DW propagation behaviour is controlled by precessional processes that leads to Walker breakdown^23^, and which in turn can be controlled by periodic structuring of the nanowires^24, 25^, the influence of additional interfacial damping and IDMI on the domain wall behaviour has not been studied in detail. Understanding the influences of both enhanced damping and IDMI on the DW dynamic behaviour relates to a fundamental physical understanding and is relevant to technological applications.

Here the objective is to understand and separate the physical mechanisms that modify and control the magnetization processes in Pt-ferromagnet nanowire structures. The contribution from the spin-Hall driven spin-current does not occur as the measurements undertaken without the injection of charge current into the structures. Since the IDMI is understood to be closely associated with the interface of the ferromagnetic layer to the Pt, but the damping enhancement depends upon the Pt thickness, then measuring the magnetization reversal behaviour in thin-film nanowire structures in which the thickness of the Pt layer was varied gives insight into the two contributions of the physical processes. By combining both experimental measurements of bilayered nanostructures and micromagnetic simulations, the DW dynamic behaviour was studied to determine the effects of the different contributions arising from interfacing Pt with the ferromagnetic layer.

## Experimental and Micromagnetic Simulation Details

**Figure 1:**
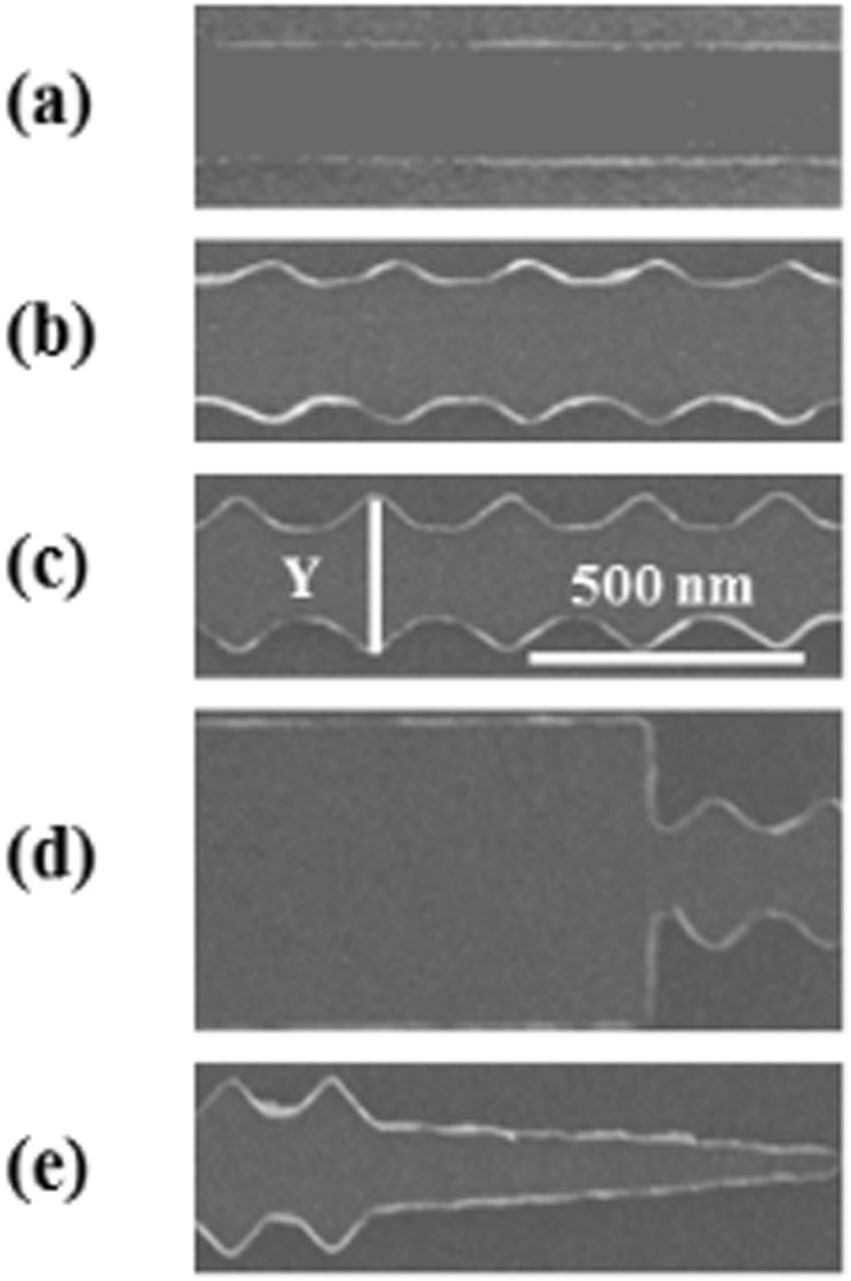
Scanning Electron Microscopy (SEM) images of nanowires with different geometries produced to study DW propagation in bilayer NiFe/Pt films. Periodic modulation placed on the nanowire edges provides pinning potential to the DW propagation. Also, pad injection and tapered shape were used to inject and annihilate DWs, respectively.

To understand the influence of the Pt layer on the DW reversal behaviour a range of nanowire geometries were patterned by electron-beam lithography, magnetron sputtering deposition and lift-off, as it is shown in Fig. 1. Various wire geometries were studied to obtain a generalised understanding of the influence of the Pt layer. Parallel-sided and fixed-angle periodic triangular featured nanowires were fabricated to provide modulation that is known to influence the DW propagation^25^. The parallel-sided nanowire has a width of 250 nm. For the modulated structures, additional periodic triangular features with amplitudes varying from 30 nm to 75 nm were patterned along both edges. A rectangular pad 7 *μ*m × 1 *μ*m was fabricated at one end of the nanowire to create and inject individual DWs. At the other end, a tapered shape was used to prevent local DWs nucleating. Bilayered thin films were grown onto Si/SiO_2_ substrates using UHV magnetron sputtering. The film stacks grown were Ni_81_ Fe_19_(10 nm)/Pt(t_*NM*_), where t_*NM*_ is the Pt layer thickness, which was varied between 0 up to 3 nm. X-ray reflectivity measurements were carried out to determine the thickness of the layers and the interfacial roughness. Magnetisation reversal was measured as a function of Pt thickness using focused magnetic-optic Kerr effect magnetometry to investigate reversal mediated by DW propagation in individual nanowires^26^. The laser spot interrogated a length of approximately 5 *μ*m on the nanowire surface and the magnetic field was applied parallel to the nanowire axis. The signal-to-noise ratio was improved by averaging many hundreds of measured field cycles.

To add physical insight and support the interpretation of the experimental magnetization reversal behaviour numerical micromagnetic calculations were performed to understand the effects of both DMI and enhanced damping upon the magnetization reversal in nanowires. The simulations were performed using the Mumax^3^ code^27^ by solving the Landau-Lifshitz-Gilbert equation given by,

1 $$\frac{\partial {\bf{m}}}{\partial T}=-{\bf{m}}\times {{\bf{H}}}_{{\rm{eff}}}+\alpha {\bf{m}}\times \frac{\partial {\bf{m}}}{\partial T},$$

where **m** is the magnetization, T = (*γ* M_*s*_)^−1^ t is the time, **H**_eff_ is the effective field and *α* the phenomenological Gilbert damping constant. The effective field can be written as follow,

2 $${{\bf{H}}}_{{\rm{eff}}}={{\bf{H}}}_{{\rm{ext}}}+{{\bf{H}}}_{{\rm{exc}}}+{{\bf{H}}}_{{\rm{ani}}}+{{\bf{H}}}_{{\rm{ms}}}+{{\bf{H}}}_{{\rm{DMI}}}$$

which includes contributions from the external field, exchange, magnetic anisotropy, magnetostatic fields and the DMI effective field, respectively. Thus to minimize the energy of the system these terms are taken into account in order to study numerically the field-driven domain wall behaviour.

The parameters used for Ni_81_Fe_19_ are: exchange stiffness constant A = 13 × 10^−12^ J/m, saturation magnetization M_*s*_ = 860 × 10^3^ A/m and zero magnetocrystalline anisotropy. The thickness of Ni_81_Fe_19_ was 10 nm and nanowire width was 250 nm for parallel-sided wires. For the nanowires with periodic triangular features, the amplitude was varied from 30 nm to 75 nm from the main edge of the nanowires. In all simulations the wire length was 5 *μ*m. The simulations were discretized into cell size of 5 × 5 × 10 nm^3^, which is justified by being comparable with the exchange length for NiFe^28^ in the x-y plane. Simulations were also performed using smaller cell sizes such as 3 nm × 3 nm. The results observed were quite similar to those obtained with 5 nm × 5 nm cells. In the simulations, transverse domain walls (T-DWs) were formed 1 *μ*m from the nanowire end. The formation of a transverse wall is consistent with previous works, although the vortex wall may have lower energy, both T-DW and vortex walls can be supported for such nanowires dimensions^29, 30^ as the energetic difference is small^31^. The magnetic field was then applied from 1 Oe up to 200 Oe in order to propagate the DW. The reversal fields were calculated for DW mediated magnetization reversal while varying the damping coefficient between 0.01 and 0.1. In addition, the IDMI was also varied from 0 up to 1 mJ/m^2^ to provide further insight on DW propagation. Thus, the DW propagation behaviour was studied as a function of both parameters. The analyses do not show any significant difference in reversal field with respect to the DW chirality.

**Figure 2:**
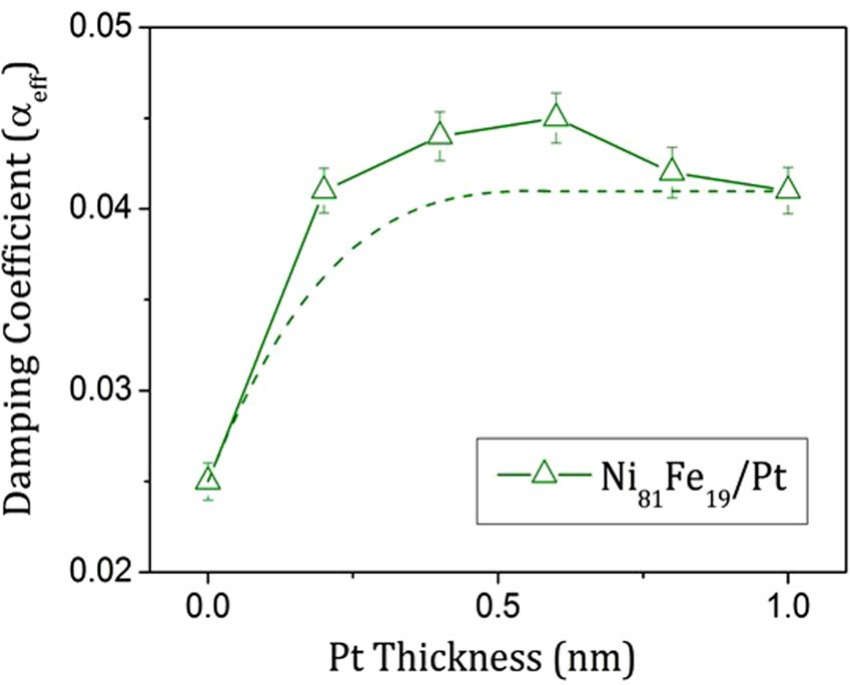
Effective damping coefficient obtained in NiFe/Pt thin films. The damping increases rapidly between 0 up to 0.6 nm Pt thickness, then above it goes down slowly. The dashed line illustrates the expected contribution to the damping from intrinsic processes.

The influence of the heavy metal layer on precessional damping in relation to layer thickness and interfacial roughness was previously studied in detail in thin films as a function of Pt thickness^3^. The precessional magnetization behaviour showed single mode damped oscillatory magnetization behaviour, from which the damping coefficient was extracted. With increasing Pt thickness the damping increased rapidly for nominal Pt thicknesses up to 0.6 nm, where the damping reaches a peak and was then reduced slightly for thicker Pt films, see Fig. 2. The analysis of the experimental thickness dependence by Azzawi *et al*.^3^ was based upon a combination of theory^6^, to explain the enhancement of the intrinsic damping, and experimental evidence for an additional structure dependent extrinsic contribution to the damping. Structural analysis indicated that the Pt layer does not provide complete surface coverage of the NiFe layer until the thickness exceeds 0.9 nm. This non-uniformity gives rise to the extrinsic damping contribution, which adds to the intrinsic enhancement of the damping, and is attributed to local variations of the *d-d* hybridisation and spin-pumping across the interface that leads to two-magnon scattering when the Pt layer is incomplete^3^. Thus, with increasing Pt thickness the damping increases initially with both intrinsic and extrinsic contributions up to a thickness of about 0.9 nm and then levels out for thicker Pt layers, where the enhanced damping is largely of intrinsic origin. The intrinsic damping is therefore expected to rise rapidly with Pt layer thickness and then plateau for greater thicknesses, as suggested by the dashed line in Fig. 2.

Modified damping as a function of Pt thickness in FM/NM bilayers films has been determined for different ferromagnetic layers such as NiFe, Co^3, 32^ and amorphous finemet films^33^ as well as for the ferrimagnet yttrium iron garnet (YIG)^34^. As the enhanced damping behaviour observed in these structures depend upon many features, including growth conditions, materials miscibilities and surface energies, the mechanism relating to the trend obtained for the damping across the Pt thickness in these materials may be similar, but the quantitative thickness dependence will not be universal. The main point to note is that the damping modifies the magnetization precession and damping and its variation with Pt thickness should be taken into account as a mechanism for altering the domain wall motion.

## Results, Analysis and Discussion

**Figure 3:**
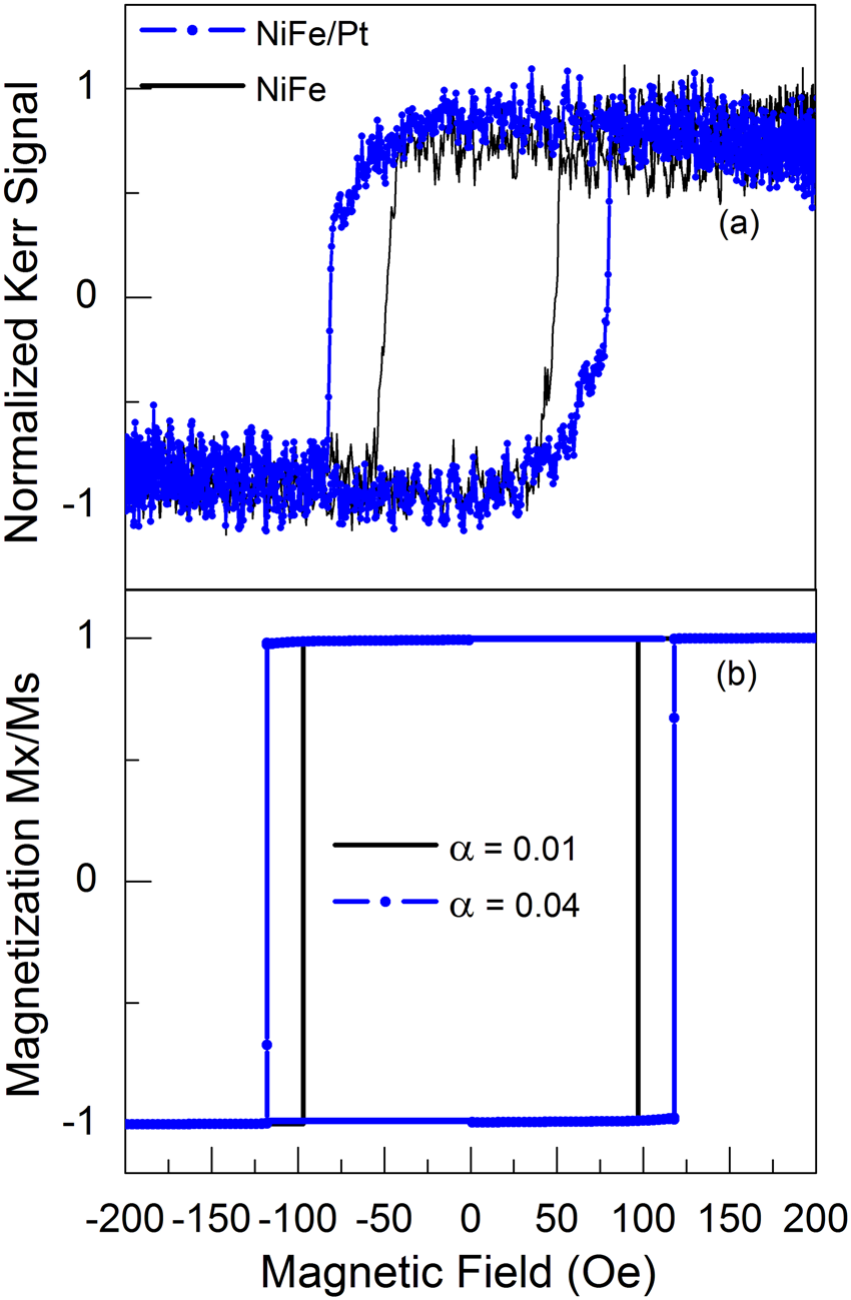
Examples of axial magnetic hysteresis behaviour for nanowires (triangular modulated edges with 75 nm amplitude). (**a**) Measured hysteresis for Py (10 nm) (continuou﻿s line) and Py(10 nm)/Pt(0.6 m) nanowires (dots). The reversal field is observed to be higher for Py(10 nm)/Pt(0.6 m) than Py(10 nm). (**b**) Simulated m﻿agnetic hysteresis for nanowires with a damping parameter of 0.01 (continuous line) and 0.04 (dots) show that increased damping provided by Pt overlayer enlarges the reversal field for larger damping.

With knowledge of the damping in thin films, the behaviour of the domain walls in nanowires was studied as a function of the Pt layer thickness using the MOKE measurements to determine the domain wall reversal field. Figure 3(a) shows the MOKE measurements for uncapped Py and for Py/Pt (0.6 nm) nanowires with fixed-angle periodic amplitude modulation of 75 nm. The reversal field needed to propagate the domain wall is observed to be larger for Py/Pt (0.6 nm) nanowire than for the uncapped Py nanowire. This indicates that the Pt capping layer plays a role on the domain wall propagation related to reversal field in the magnetization reversal process. Figure 3(b) shows examples of hysteresis loops from micromagnetic simulations of similar edge-modulated NiFe nanowires with damping values of 0.01 and 0.04 respectively. In the experiments the lowest observed damping was 0.025 and highest was 0.045, the simulations were performed for a wider range of values between 0.01 and 0.1, in order to more fully explore the effect of the modified damping upon the reversal field and the domain wall propagation. As can be seen in the Fig. 3(b), the reversal field is higher for the damping coefficient of 0.04 than for the value of 0.01, which agrees with the experimental hysteresis obtained by MOKE measurements and supports the supposition that the enhanced damping from the Pt overlayer affects the DW reversal process, although for a thin incomplete Pt layer, the formation of Pt islands on the NiFe could create inhomogeneities that add extra pinning to the DW propagation. However, as will be shown the trend of increased reversal field continues for Pt layers that are thicker and provide complete coverage of the NiFe layer.

**Figure 4:**
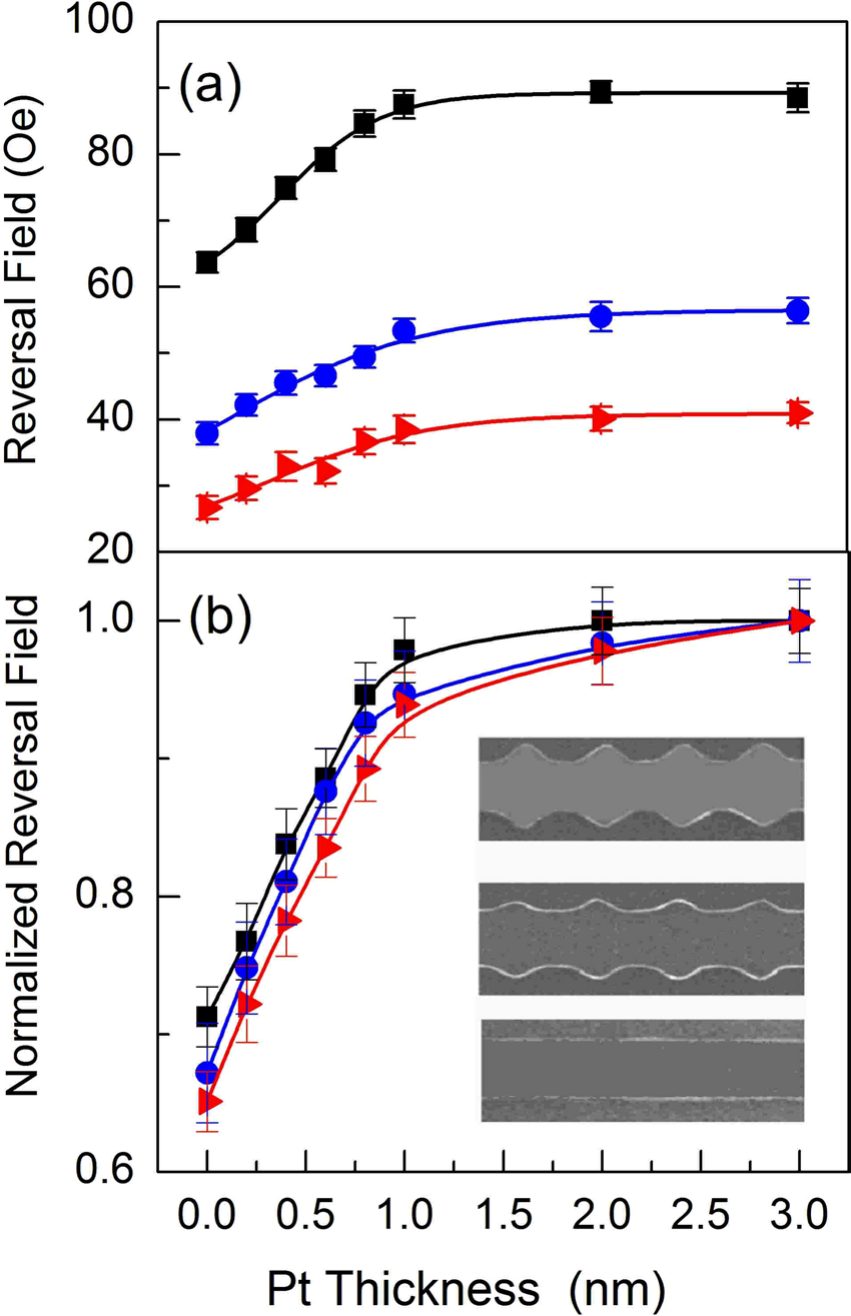
Reversal field as a function of Pt thickness. In (**a**), for different periodic edge-modulated nanowires the reversal field goes up rapidly from 0 nm to 1 nm Pt thickness, for thicker Pt the reversal field is roughly constant. In (**b**), the normalized reversal field shows that the behaviour as a function of Pt thickness is, indeed, independent of the nanowire geometry. Insert, Scanning electron microscopy (SEM) images of nanowires show the different geometries used to studied DW propagation in NiFe with a Pt overlayer. The triangles, dots and squares observed in (**a**) and (**b**) are related to parallel sided and periodic modulated 30 and 75 nm amplitude, respectively.

To further understand and generalise the effect of the heavy metal Pt overlayer on the DW propagation in Ni_81_Fe_19_ nanowires the reversal field was measured as a function of Pt layer thickness up to 3 nm. The experimentally measured field required for magnetization reversal mediated by DW propagation in the three different nanowire geometries is shown as a function of Pt capping layer thickness in Fig. 4(a). For the parallel-sided wire (see triangles), the reversal field increases monotonically with increasing Pt thickness between 0 up to 0.8 nm thickness. For thicknesses above 0.8 nm, the reversal field rises more slowly towards a plateau. For the triangular modulated nanowires with 30 nm and 75 nm amplitude features (see dots and squares respectively), the reversal field has a similar trend to that observed in the parallel-wire sided as a function of Pt thickness, but the reversal fields are higher and the increase in the reversal field is larger for the larger amplitude edge modulation.

To assess the generalised role of the Pt capping layer on the reversal field the measured reversal fields for all three nanowire geometries were normalized to the reversal field at highest Pt thickness, see Fig. 4(b). The normalized reversal fields show very similar behaviour, with an initial linear increase in reversal field up to approximately 0.8 nm Pt thickness, followed by a slower approach towards a constant value. This trend is largely independent of the details of the nanowire geometry, which suggests, therefore, that the addition of a Pt layer of thickness up to 1 nm plays a substantial role in modifying the magnetization reversal process.

The dependence of the reversal field on the Pt thickness may be explained by considering first the change in damping. In continuous film measurements, the damping is enhanced with Pt thickness up to 0.6 nm, due to both increases in intrinsic and extrinsic mechanisms in this Pt thickness range. The effective damping peaks and falls slightly for thicker Pt. Significantly, structural analysis indicates that due to interfacial roughness the Pt forms a fully continuous layer for nominal thickness beyond approximately 0.9 nm, where analysis suggests the enhanced damping is largely of intrinsic origin, associated with *d-d* hybridisation and spin-pumping across the Pt-FM interface^3^. Thus it is suggested that the intrinsic damping increases up to approximately 0.9-1.0 nm and then becomes constant. It is interesting therefore, that for DW reversal in the nanowires the reversal field increases linearly with Pt thickness up to approximately 0.8−1.0 nm, which may suggest that the DW behaviour is sensitivity only to the intrinsic component of the damping.

**Figure 5:**
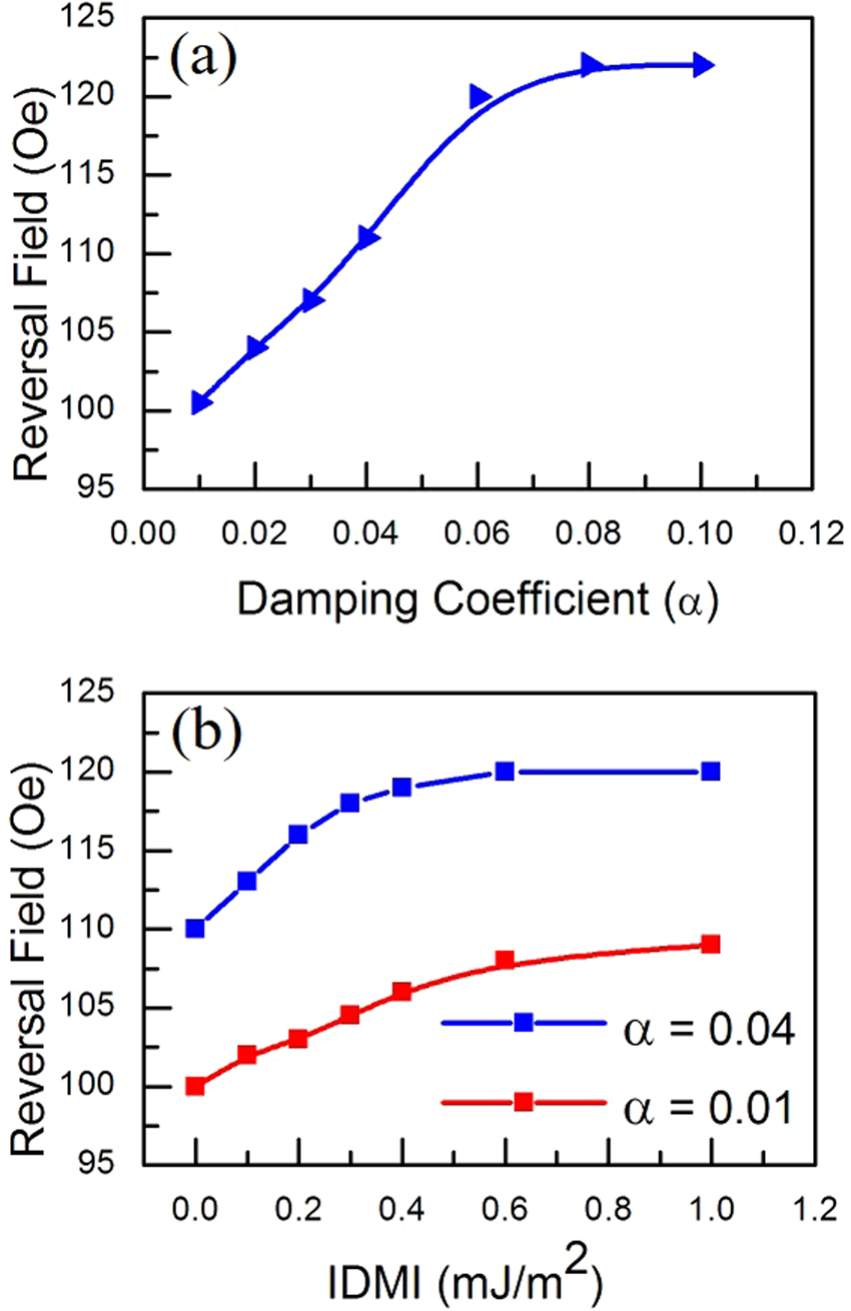
Reversal field calculated from hysteresis loops obtained in the micromagnetic simulations. By varying the damping the reversal field goes up, reaches a plateau, which is consistent with experimental data observed in Fig. 4(a). Simulations were also performed varying IDMI using two damping values. Both the reversal fields as a function of IDMI show a smaller influence on the DW propagation, tho﻿ugh for higher damping the IDMI has a more important contribution to the reversal field strength.

This DW motion dependence upon intrinsic damping is supported, first by micromagnetic analysis, see Fig. 5(a), which shows an increase in the reversal field for a nanowire as a function of increasing intrinsic damping and secondly, it has been explained elsewhere that for energetic reasons extrinsic, or indirect relaxation will be inhibited over length-scales comparable with the domain wall width^35^. The reversal fields calculated in the micromagnetic simulations were higher than the values observed experimentally. Simulations performed with 3 × 3 nm cell size also showed similar but slightly lower values than for the 5 × 5 nm cell simulations. The discrepancy between modelling and experiment may be attributed to various inherent limitations that can occur in physical models. Here, the simulations were at zero temperature, which is well known to increases the reversal field. Furthermore, the simulations took widely used values of the physical parameters for NiFe, such as the exchange constant, but these may differ slightly from the actual values in the samples and finally, there is some difference in the detailed geometry for the samples and the simulations, as the samples will have some edge roughness from the lithography and the simulated structures are constructed of square cells.

The micromagnetic simulations show a near linear increase in the reversal field with increasing damping before reaching a plateau. Thus an increase in the intrinsic damping may explain much of the linear increase and plateau behaviour observed for the reversal field. Also, for larger damping the relaxation of magnetization towards the equilibrium tends to be faster than for lower damping reducing the torque on the domain wall. Thus the magnetic field must be increased to provide DW magnetization reversal when the damping becomes larger.

However, to understand the role of other mechanisms, and specifically interfacial DMI, that can also occur at the NM/FM interface and affect the reversal behaviour, detailed micromagnetic simulations were performed. In these simulations nanowires were modelled with selected damping values and the strength of the IDMI was varied over a range consistent with previous experimental observations^36, 37^ which suggest that the IDMI strength has some dependence on the ferromagnetic and heavy metal layer thickness. Thus, the IDMI effect was explored using two values of damping 0.01 and 0.04 with IDMI varying from 0 up to 1 mJ/m^2^.

Figure 5(b) summarises the results of these simulations for an edge-modulated nanowire. For an intrinsic damping coefficient of 0.01, the reversal field increases monotonically as a function of increasing IDMI. The percentage increase between D = 0 and 1 mJ/m^2^ is ≈8%. For higher damping of 0.04 the IDMI has a larger effect on the magnetization reversal, increasing by ≈14% from DMI = 0 to DMI = 1 mJ/m^2^. This larger increase of the reversal field might be also explained due an increase in the DW inertia arising from combination of the IDMI and the damping^38–40^. As demonstrated analytically^40^ considering a ferromagnetic wire modelled as a one dimensional classical spin chain, the solution of the Landau-Lifshitz-Gilbert equation results in the well-known expression for the domain wall width,

3 $${{\rm{\Delta }}}_{0}^{wall}=\sqrt{\frac{A}{k}},$$

where A is the exchange coefficient, k the anisotropy constant and ${{\rm{\Delta }}}_{0}^{wall}$ the domain width in the absence of DMI. On the other hand, taking into account the DMI influence, equation (3) becomes,

4 $${{\rm{\Delta }}}^{wall}=\sqrt{\frac{A}{2Ak-{D}^{2}}},$$

in which ${{\rm{\Delta }}}^{wall}$ is the domain wall width including DMI, where *D* is the DMI constant and, due to the factor ${D}^{2}$ appearing, indicates that the DW width does not depend on the sign of the DMI. Therefore, from the equation (4) the domain wall width is enlarged leading to higher inertia when comparing to the case without DMI. As a consequence, it requires a larger magnetic field to propagate the DW and produce magnetization reversal.

These results suggest that while the damping plays a major role in increasing the DW propagation field, across the range of Pt thicknesses, the role of the IDMI becomes more significant as the damping increasing and thus the influence of both on the magnetization reversal become more significant for thicker Pt layers.

It is important to note in the simulations that no static modifications of the domain wall type or chirality were observed in the DW nucleation process at zero magnetic field for different IDMI and damping values. Also, when an increasing magnetic field was applied transitions from transverse domain wall to vortex domain wall were observed in all cases, but the domain wall mobility and the onset of Walker breakdown varied depending on the strength of the IDMI and the magnitude of the damping.

**Figure 6:**
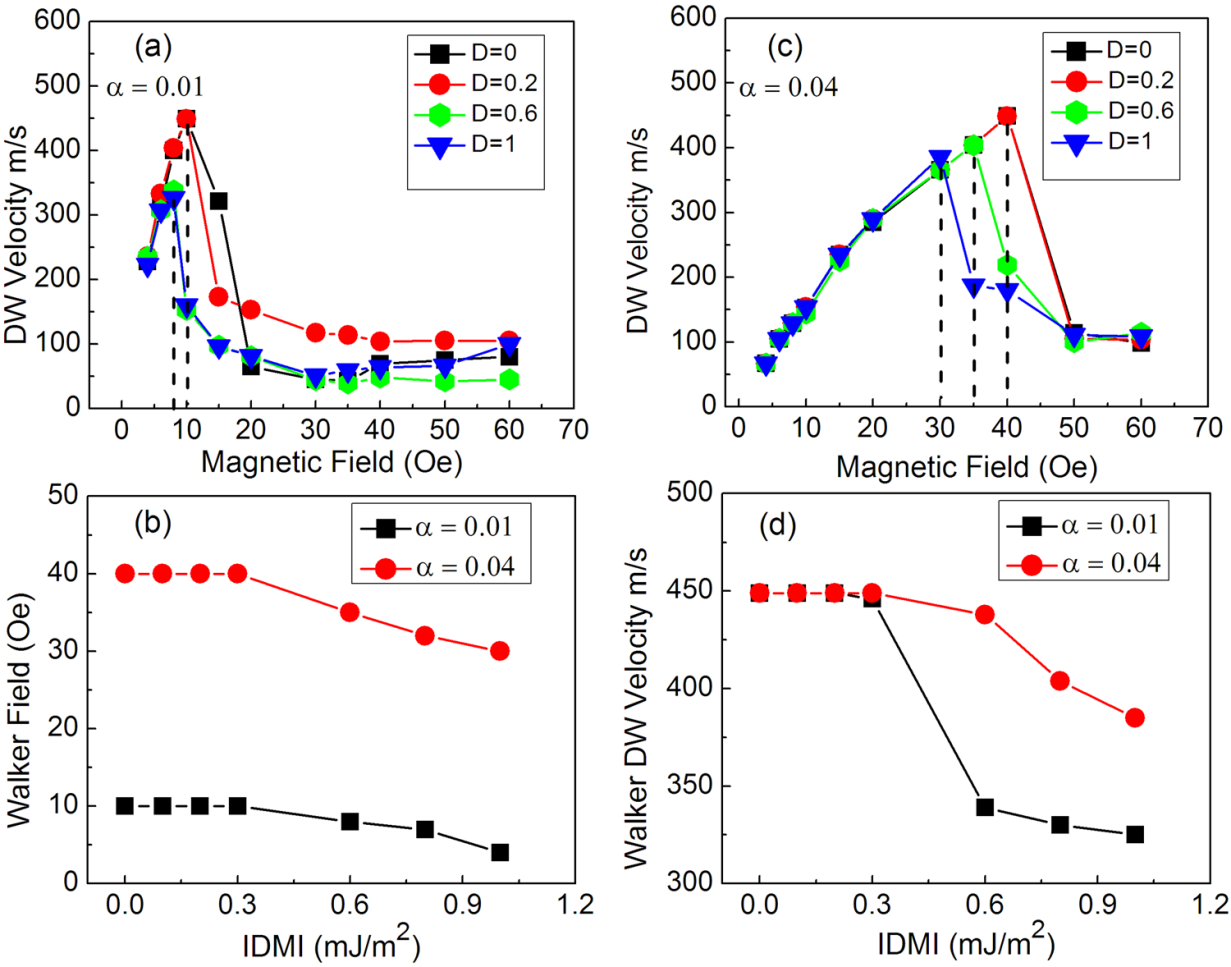
DW velocity calculated in parallel sided nanowires for various IDMI and damping 0.01 (**a**) and 0,04 (**c**). For both the damping values the velocity undergoes a reduction beyond the Walker Field threshold. The Walker F﻿ield is higher for damping of 0.04 than 0.01. However, the IDMI affects both the Walker field (**b**) and DW velocity (**d**), which for IDMI beyond 0.3 (mJ/m^2^) are decreased.

To understand the combined contributions to the magnetization reversal mediated by DW propagation in nanowires the DW velocity was investigated as a function of both the damping and IDMI. This is relevant to applications based on DWs as a reliable magnetization reversal process requires controllable velocity and a stable DW structure. Figure 6(a) and (c) show the field dependence of the DW velocity for a parallel-sided nanowire, obtained from simulations, for a range of IDMI values and for low and high damping respectively. The DW velocity increases with field and reaches a peak velocity with the onset of Walker breakdown, which occurs at the Walker Field (WF), above which the velocity falls at higher fields. As expected from precessional dynamics, the Walker breakdown occurs at a lower field for the lower damping, with higher damping leading to an extended field range of increasing domain wall velocity, but no change in the peak velocity. This also suggests that for large damping the DW has an increased inertia, which slows the DW motion at low fields. The dependence of domain wall velocity related to damping was previously discussed in refs 41 and 42 where the Walker Field was derived from the LLG equation and found to be proportional to the damping, and the domain wall mobility was predicted to depend on *γ*.Δ/*α* (where Δ is the wall width), which gives support to the simulations.

For in-plane magnetized nanowires increasing the IDMI leads to the onset of Walker breakdown at a lower field, as indicated by the dashed lines in Fig. 6 (a) and (c) and which leads to a lower peak velocity. Also, the effect of the IDMI on both domain wall velocity and Walker field has been extracted from micromagnetic simulations for low and high damping. With increasing IDMI for high (0.04) and low damping (0.01) the WF and the peak DW velocity were observed to fall for this micromagnetic implementation of DMI, as shown in Fig. 6(b) and (d). For values of IDMI (up to 0.3 mJ/m^2^) the Walker field and DW velocity at Walker field are constant. Above this D value it is observed that both the WF and the velocity decrease in a range of IDMI that is physical reasonable for the NiFe/Pt system. Also, it is noted that the WF undergoes a larger change in response to the IDMI value when the damping is higher, but the opposite is observed for the peak DW velocity, Fig. 6(d). This indicates that both the Walker field and velocity are modified when interfacial effects such as damping and IDMI occur simultaneously.

**Figure 7:**
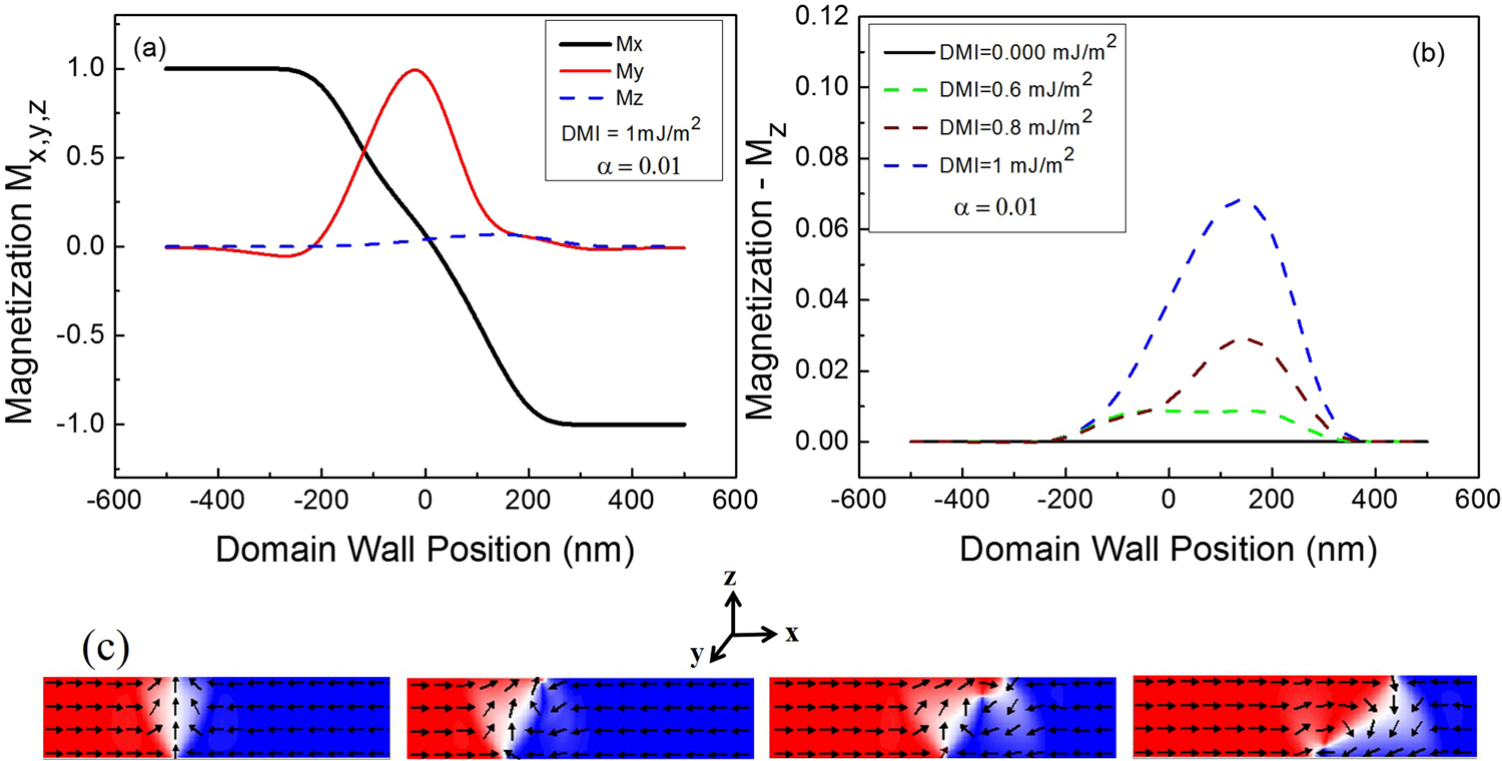
Magnetization component calculated along of the DW position in the nucleation process for damping equal 0.01. In (**a**), the M_*x*_ magnetization component varies from 1 to −1 due the magnetic moments head to head around the DW. The M_*y*_ magnetization component has a positive value as expected for transverse domain wall up. Interestingly, the M_*z*_ component, see dashed blue line, emerges for a DMI value of 1 mJ/m^2^. In (**b**) these magnetization components were calculated for different values of DMI and it is shown that the M_*z*_ magnetization component increases as the DMI becomes larger. The continuous line shows the absence of M﻿﻿_*z*_ magnetization component without DMI. In (**c**), snapshots shows the transitions from transverse domain wall up to down mediated by core vortex nucleation.

This effect may be explained by the local re-orientation of the spin-structure at the interface due to the influence of the IDMI on the magnetization orientation at the interface, as indicated by the magnetization components extracted from micromagnetic simulations as a function of domain wall position in the nucleation process, see Fig. 7(a) and (b). The magnetization components M_*x*_, M_y_ and M_*z*_ have directions along the wire axis, transverse and perpendicular, respectively. M_*x*_ varies from positive to negative values, as expected for head-to-head domains separated by a domain wall. The M_*y*_ is predominantly positive as the simulations showed a transverse domain wall with up polarization. The most interesting behaviour lies in the M_*z*_ component, which is identified by the dashed blue line and is shown in more detail in the Fig. 7(b). As the IDMI is increased a magnetization component out-of-plane emerges along the domain wall width and becomes larger as the IDMI increases. Without IDMI this M_*z*_ component is absent, see the continuous line in Fig. 7(b). Here the M_*z*_ magnetization component was predominantly positive for positive values of the DMI.

For in-plane magnetized nanowires the transitions from T-DW to V-DW (vortex domain wall) occurs by the nucleation of a magnetic component out-of-plane that usually energetically favours vortex-core nucleation^43^, as it is shown in the representative snapshots, see Fig. 7(c). The IDMI may act to provide a component of the magnetization out-of-plane that aids the nucleation of the DW vortex core. Therefore, for nominally in-plane magnetized nanowires the IDMI must be highly controlled to avoid the loss of DW velocity. Simulations using negative IDMI values were also performed in order to observe any different behaviour regarding the domain wall velocity. As it is observed for positive IDMI values the onset of Walker breakdown occurs at a lower field and hence the peak velocity is reduced when increasing the IDMI amplitude. However, for a given IDMI amplitude an asymmetric behaviour is obtained for negative IDMI where the peak velocity is lower when comparing to equivalent positive IDMI values.

The findings here showing the influence of DMI on the domain wall velocity for in-plane magnetized (IMA) nanowires are quite different from that reported elsewhere for systems with out-of-plane magnetization (PMA), where the domain walls are stabilized from Bloch to Néel states depending upon the amplitude and sign of the interfacial DMI^44^. The domain wall motion in such perpendicularly magnetized nanowires has higher domain wall mobility with DMI and increased stability, and Walker breakdown is pushed to higher driving fields^14^. The robust domain wall motion found in perpendicularly magnetized nanowires allows faster reversal of the magnetization as the DW angle is fixed under strong DMI^14, 45^. The domain wall velocity reaches the peak velocity without precessional breakdown and maintains the variation of the angle almost constant for a range of magnetic fields, avoiding domain wall rotation around the wire axis and hence transitions from Néel to Block walls. In contrast, for in-plane magnetized nanowires with DMI the domain wall does not avoid transitions from transverse to vortex domain wall when propagating under a magnetic field that is higher than the Walker field threshold. Simulations indicate that the domain wall translates and rotates around the nanowire axis and it leads to back-and-forth motion slowing down the domain wall velocity for larger magnetic fields.

## Conclusion

Experimental results and micromagnetic analyses on domain wall magnetization behaviour show the influence of the different phenomenon occurring at the interface in NM/FM bilayers, where the ferromagnetic layer is magnetized in-plane and coupled to a heavy metal, in this case Pt. The influence of the Pt layer thickness on the magnetization reversal was investigated. By studying the magnetization reversal in parallel-sided and edge modulated Ni_81_Fe_19_/Pt nanowires, where the Pt thickness was varied from 0 nm up to 3 nm, both the damping and IDMI were explored as sources to understanding the magnetization reversal behaviour. Magnetization reversal measurements showed that the reversal field increases as a function of Pt thickness between 0 up to 1 nm, increasing rapidly up to 0.8 nm and then tending to a plateau for Pt thickness up to 3 nm. The same trend in the reversal behaviour was observed in parallel-sided and modulated nanowires edges indicating that the influence of the heavy metal layer does not depend on the details of the nanostructure shape.

The details of the influence of intrinsic damping and the IDMI arising from the Pt capping layer were analysed by micromagnetic analysis in order to understand their respective effects upon the magnetization reversal. The DW de-pinning and propagation depends more strongly on the increased damping, but the IDMI plays an increasing role on the magnetization reversal as the damping increases. Furthermore, both the damping and IDMI were observed to significantly alter the DW velocity and the Walker field. Increasing damping shifts the peak velocity and Walker field to higher magnetic fields, while inclusion of IDMI reduces both Walker field and velocity.

The combination of a heavy metal layer with large spin-orbit with a ferromagnetic thin-film can influence the magnetization behaviour of the ferromagnetic system via both the effects of Dzyaloshinskii-Moriya interaction across the interface between the heavy metal and ferromagnet and by the increased damping arising from hybridisation and spin-pumping across the FM/NM interface. For DW dynamic processes regarding to velocity and Walker Field, the manipulation of both damping and weak IDMI may aid the control of DW behaviour and help provide further control in applications and enhancement of device performance.
